# Adolescent cannabis and tobacco use and educational outcomes at age 16: birth cohort study

**DOI:** 10.1111/add.12827

**Published:** 2015-02-07

**Authors:** Alexander I. Stiby, Matthew Hickman, Marcus R. Munafò, Jon Heron, Vikki L. Yip, John Macleod

**Affiliations:** ^1^ School of Social and Community MedicineUniversity of BristolBristolBS8 2PSUK; ^2^ MRC Integrative Epidemiology Unit, UK Centre for Tobacco Control Studies and School of Experimental PsychologyUniversity of BristolBristolUK; ^3^ Avon Longitudinal Study of Parents and Children, School of Social and Community MedicineUniversity of BristolBristolUK

**Keywords:** ALSPAC, cannabis use, cotinine, education, English, GCSE, mathematics, school dropout, smoking

## Abstract

**Aims:**

To investigate the relationship between cannabis and tobacco use by age 15 and subsequent educational outcomes.

**Design:**

Birth cohort study.

**Setting:**

England.

**Participants:**

The sample was drawn from the Avon Longitudinal Study of Parents and Children; a core sample of 1155 individuals had complete information on all the variables.

**Measurements:**

The main exposures were cannabis and tobacco use at age 15 assessed in clinic by computer‐assisted questionnaire and serum cotinine. The main outcomes were performance in standardized assessments at 16 [Key Stage 4, General Certificate of Secondary Education (GCSE)] in English and mathematics (mean scores), completion of five or more assessments at grade C level or higher and leaving school having achieved no qualifications. Analyses were sequentially adjusted for multiple covariates using a hierarchical approach. Covariates considered were: maternal substance use (ever tobacco or cannabis use, alcohol use above recommended limits); life course socio‐economic position (family occupational class, maternal education, family income); child sex; month and year of birth; child educational attainment prior to age 11 (Key Stage 2); child substance use (tobacco, alcohol and cannabis) prior to age 15 and child conduct disorder.

**Findings:**

In fully adjusted models both cannabis and tobacco use at age 15 were associated with subsequent adverse educational outcomes. In general, the dose–response effect seen was consistent across all educational outcomes assessed. Weekly cannabis use was associated negatively with English GCSE results [grade point difference (GPD), –5.93, 95% confidence interval (CI) = –8.34, –3.53] and with mathematics GCSE results (GPD, –6.91, 95% CI = –9.92, –3.89). Daily tobacco smoking was associated negatively with English GCSE (GPD, –11.90, 95% CI = –13.47, –10.33) and with mathematics GCSE (GPD, –16.72, 95% CI = –18.57, –14.86). The greatest attenuation of these effects was seen on adjustment for other substance use and conduct disorder. Following adjustment, tobacco appeared to have a consistently stronger effect than cannabis.

**Conclusions:**

Both cannabis and tobacco use in adolescence are associated strongly with subsequent adverse educational outcomes. Given the non‐specific patterns of association seen and the attenuation of estimates on adjustment, it is possible that these effects arise through non‐causal mechanisms, although a causal explanation cannot be discounted. © 2015 Society for the Study of Addiction

## Introduction

Cannabis use, particularly among young people, is still relatively common 1, 2, 3. UK cannabis use has been reportedly declining since its peak, although 2012/13 figures estimate that 30.9% of 16‐24 year olds have ever used cannabis and 13.5% have smoked cannabis in the last year 4. Various adverse psychosocial outcomes have been reported to be associated with cannabis use; however, the causal basis for these associations is often unclear. Lower educational attainment, for example, is associated consistently with higher use of cannabis. Evidence that this association is causal, such that preventing cannabis use among young people would increase their educational attainment, would have important implications for policy. A recent co‐twin control study found that cannabis does not cause adverse education outcomes, but both traits are influenced by the same family environmental factors 5. The available population‐based evidence is exclusively observational, reflecting the practical and ethical difficulties inherent in an experimental approach. This situation is common in aetiological epidemiology, and several strategies have been devised to guide causal inference in observational data 6. These strategies include consideration of evidence for non‐causal associations such as those arising through confounding, measurement approaches that reduce the potential for bias and the use of longitudinal data to establish direction of causality. A further, perhaps neglected criterion for causality is specificity of association 7. In general, non‐specific associations are less likely to be causal 8.

In a large population‐based prospective observational birth cohort study we investigated the effects of cannabis use by age 15 on subsequent educational outcomes. We examined evidence for confounding by adjusting for multiple possible confounding factors in multivariate models. We used linkage to independent administrative data to ascertain educational outcomes to minimize the risk of measurement bias related to self‐report. We also adjusted effect estimates for educational attainment prior to cannabis use to address the issue of reverse causation. Finally, we considered the issue of specificity of association by investigating the effects of tobacco use on the same educational outcomes. We investigated the effects of both biologically verified and self‐reported tobacco use, again to consider evidence for possible bias.

## Methods

### Data

The core sample of the Avon Longitudinal Study of Parents and Children (ALSPAC) includes 14 541 women who were expecting to deliver infants between 1 April 1991 and 31 December 1992 in the former county of Avon, UK. ALSPAC parents and children have been followed‐up regularly since recruitment 9. Ethical approval for the study was granted by the ALSPAC Law and Ethics Committee and the local research ethics committee. Full details about the ALSPAC study and design are described elsewhere (http://www.bristol.ac.uk/alspac).

### Outcome variables

The outcome variables used were standardized compulsory examination results at Key Stage 4, known as General Certificate of Secondary Education (GCSE) results; these were all from the National Pupil Database (NPD) 10. The variables investigated were: English GCSE results (per cent), Mathematics GCSE results (per cent), did not gain grade C or above in five or more GCSEs and gaining no GCSE passes. GCSE grades in English and Mathematics were converted into a percentage from a letter grade by using the median percentage in each grade category. Grades were available in nine categories and range from ‘A Star’ to G and U (ungraded/unclassified), and encompass 10% per grade point category, except U, which corresponds to 20%. We used the outcome not gaining 5 or more GCSE results at grade C because 5 or more GCSEs at grade C is the standard level for entry into post‐16 education, therefore not achieving this will probably mean not continuing to college or sixth form. Gaining no GCSE passes would infer dropping out of school at 16 with no qualifications. These were all measured at approximately 16 years of age (the standard age for taking GCSEs and the end of compulsory education in England).

### Exposure variables

The exposure variables considered were measured in the clinic using computer‐based questionnaires, as follows: cannabis use (never, ever), cannabis use frequency (never, non‐weekly, weekly) and a Cannabis Abuse Screening Test (CAST) score of 4 and above (no, yes) 11. The CAST score is a standard set of questions to measure an individual's use of cannabis. A score of 4 or more was used as a measure of cannabis ‘abuse’, also known as problematic use (i.e. use which could lead to detrimental health or social consequences) 11.

Self‐reported tobacco use (never, less than daily, daily) and tobacco use assessed by measuring serum cotinine biomarker measures were used (Appendix 1). Participants were classified as smoker/non‐smoker using the cut‐off of 9.5 ng/ml blood 12, 13. As cotinine has a half‐life of around 24 hours, this categorization in effect classifies individuals as daily smokers versus non‐daily or non‐smokers.

### Covariates

Covariates were included on the basis of either previous evidence that they were associated with both substance use and educational outcomes or theoretical considerations suggesting that they may confound an association between these. Covariates were grouped into proximate and distal determinants. The distal determinants can have an inter‐relationship with the more proximate determinants, and therefore need to be ordered in this way for multivariable analysis 14. Covariate models included measures within broad models; these models were grouped into maternal substance use, demographics, previous educational attainment and child behaviours, such as substance use and conduct disorder (see Appendix S1 for full explanation of the covariate models).

### Sample derivation

The starting sample for analysis in the ALSPAC cohort was 14 062 singletons and twins; these were born live. The sample's exposure was measured in the clinic; of the 14 062 individuals, 9985 (71%) of these live‐born children were invited to the ‘Teen Focus Three’ clinic (TF3) at approximately 15.5 years of age 9; 5190 (52%) of these children attended TF3. Of those who attended the clinic, 5137 (99%) answered questions about cannabis use, 4802 (92.5%) answered the CAST and 4433 (85.4%) answered tobacco use questions. Cotinine was measured in 3350 (64.6%) individuals. GCSE data in the NPD are available only for pupils attending state schools, and not all data items are complete for these individuals. Descriptive characteristics of the data presented are also included (Table  1a and b).

**Table 1:** (a) Descriptive characteristics for educational outcomes and substance use exposures using the imputed data set.

*Outcome*							
		*English GCSE*			*Mathematics GCSE*		
*Exposure*		*n*	*Mean*	*SE*	*n*	*Mean*	*SE*
Cannabis use	Never	3277	71.34	0.24	3198	70.46	0.28
	Ever	1098	68.53	0.42	1066	65.04	0.48
	Total *n*	4375	70.63	0.20	4264	69.11	0.24
Frequency of cannabis use	Non‐smoker	3958	70.77	0.21	3866	69.54	0.26
	Non‐weekly smoker	297	71.18	0.75	288	65.71	0.87
	Weekly smoker	120	64.83	1.28	110	62.64	1.49
	Total *n*	4375	70.63	0.20	4264	69.11	0.24
CAST score of 4 or above	No	3982	71.10	0.21	3876	69.76	0.25
	Yes	105	65.95	1.40	102	60.88	1.53
	Total *n*	4087	70.97	0.21	3978	69.53	0.25
Smoking status	Non smoker	2996	72.19	0.24	2914	71.63	0.29
	Non‐daily smoker	472	69.40	0.59	451	66.08	0.65
	Daily smoker	292	60.29	0.81	293	54.91	0.95
	Total *n*	3760	70.92	0.22	3658	69.61	0.27
Cotinine‐assessed smoking status	No	2572	71.47	0.26	2496	70.71	0.31
	Yes	255	60.75	0.88	251	55.68	1.04
	Total *n*	2827	70.50	0.25	2747	69.33	0.31
(b) Descriptive characteristics for educational outcomes on substance use exposures using the imputed data set.							
		*Not gaining 5+ C+ grade GCSEs*			*School dropout*		
*Exposure*		*n (yes)*	*n (no)*	*% (yes)*	*n (yes)*	*n (no)*	% (Yes)
Cannabis use	Never	671	2631	20.32	33	3268	1.00
	Ever	352	782	31.04	31	1102	2.74
	Total *n*	1023	3413	23.06	64	4370	1.44
Frequency of cannabis use	Non‐smoker	893	3105	22.34	47	3950	1.18
	Non‐weekly smoker	71	233	23.36	7	297	2.30
	Weekly smoker	59	75	44.03	10	123	7.52
	Total *n*	1023	3413	23.06	64	4370	1.44
CAST score of 4 or above	No	856	3166	21.28	46	3975	1.14
	Yes	52	64	44.83	10	106	8.62
	Total *n*	908	3230	21.94	56	4081	1.35
Smoking status	Non‐smoker	551	2468	18.25	28	2990	0.93
	Non‐daily smoker	116	362	24.27	4	474	0.84
	Daily smoker	196	123	61.44	21	297	6.60
	Total *n*	863	2953	22.62	53	3761	1.39
Cotinine‐assessed smoking status	No	513	2080	19.78	27	2565	1.04
	Yes	155	122	55.96	15	262	5.42
	Total *n*	668	2202	23.28	42	2827	1.46

CAST = Cannabis Abuse Screening Test; GCSE = General Certificate of State Education; SE = standard error.

### Analysis

Linear or logistic regression was used as appropriate. Analysis was run on the complete case and imputed data; the results in this study have been extracted from the imputed data in order to increase the power of the findings. All analyses were run on Stata version 12 15. Covariates were included on theoretical grounds, and they were tested for their impact on our parameter estimates by using the likelihood ratio test for the non‐imputed data and Stata's *testparm* function on the imputed data. The likelihood ratio test compares the fit of the covariate model to the previous model; if there is a difference, then the less restrictive model (the model with more covariates) is said to fit the data better than the more restrictive model 16.

### Data completeness

We conducted a complete case analysis, based on 1155 individuals with complete data on self‐reported substance use and serum cotinine measured in the tier three clinic. We compared estimates of effects between the imputed and complete case in order to assess any bias that may have been introduced through imputing the sample.

### Multiple imputations

To mitigate against loss of power resulting from reduced sample size and investigate possible bias related to missing data, we used multiple imputation of exposures and covariates using the ice function in Stata version 12 using the missing‐at‐random assumption 15, 17. We imputed non‐complete covariates for individuals who had missing data for a covariate and had data on the exposures. The data source on which we are imputing was from self‐reported questionnaires. The prediction equation used all other associated covariates in order to impute the missing values. Twenty imputed data sets were created, as recommended by Sterne *et al*. 18; analysis on this output file used the mim function in Stata.

## Results

### Assessment of confounding

Maternal substance use, lower social position, poorer educational performance at Key Stage 2 and children's use of other substances were associated positively both with cannabis and tobacco use at age 15 (Table S1) and with poorer educational outcomes at age 16 (Table S2).

We found that previous educational attainment was associated with current educational attainment (Table S2). We also found that previous English assessment prior to age 11 [Key Stage 2] was associated with a CAST score above 4, daily smoking of tobacco and cotinine‐assessed smoking status. Previous Mathematics assessment prior to age 11 (Key Stage 2) was associated with cannabis use, CAST score above 4, tobacco use and cotinine‐assessed smoking status (Table S1). Therefore, in order to reduce the problem of reverse causation we included previous educational attainment as a covariate in our model. Previous educational attainment is measured at a time‐point prior to initiation of these substances; high levels of attenuation within this covariate model would indicate a problem with reverse causation.

### Association between cannabis use and educational outcomes

In the univariable analyses, using imputed covariates, cannabis use is associated with lower grade point difference (GPD) in English GCSEs in an approximately dose–response fashion (Table 2). Problematic cannabis use as assessed by CAST is similar to those seen with weekly cannabis use [CAST score: –5.15 GPD, 95% confidence interval (CI) = –7.69, –2.60; weekly cannabis use: –5.93 GPD, 95% CI = –8.34, –3.53]. Adjustment for maternal substance use, social position and prior educational attainment all attenuate these estimates; the greatest attenuation is seen for prior educational attainment and on adjustment for child behaviour. Within these two categories the greatest attenuation is occurring from Key Stage 2 Mathematics and daily smoking in the previous education and child behaviour models, respectively (Table S6). After attenuation there is still evidence of a moderate effect of cannabis on education at age 16.

A similar pattern of association is seen in relation to the effects of cannabis use on attainment in Mathematics at GCSE; however, effects are generally of a greater magnitude (CAST score: –8.88 GPD, 95% CI = –11.98, –5.78) (Table 3). In contrast to attainment in English, the association of cannabis use on attainment in Mathematics remains post‐adjustment for maternal substance use, social position and prior educational attainment (CAST score: –3.52 GPD, 95% CI = –5.81, –1.23]. The greatest attenuation is seen in the ‘child behaviour’ model. Within this model, the greatest attenuation is occurring from the daily tobacco smoking covariate (Table S7).

Similar patterns of association are repeated in relation to the apparent effects of cannabis use on non‐completion of five or more GCSEs at grade C or above (table 4) and school dropout (Table 5). In the univariable analysis, weekly cannabis use is associated with higher odds of not achieving 5 or more grade C results at GCSE [odds ratio (OR) = 2.74, 95% CI = 1.93, 3.88] and higher odds of leaving school with no GCSE passes (school dropout) (OR = 6.83, 95% CI = 3.37, 13.85). The greatest attenuation occurs within the ‘prior educational attainment’ and the ‘child behaviour’ models. Within these models, the greatest attenuation occurs from the addition of Key Stage 2 Mathematics and being a daily smoker in the education and child behaviour models, respectively.

### Association between tobacco use and educational outcomes

In the univariable analyses, using imputed covariates, tobacco use at age 15 was associated with lower GPD in both English (Table 2) and Mathematics GCSE (Table 3). Tobacco use was also associated with lower odds of achieving at least 5 grade C passes at GCSE (Table 4) and greater odds of leaving school with no GCSE passes (school dropout, Table 5). Again, a broadly dose–response pattern was seen, with effects of cotinine‐verified tobacco use being similar to those of daily smoking. Apparent effects were generally stronger and of greater magnitude than those of cannabis use. The greatest attenuation of effects was seen on adjustment of ‘prior educational attainment’ and ‘child behaviour’ models. In contrast to the effects of cannabis use, the effects of tobacco use on attainment in GCSE Mathematics (–6.70 GPD, 95% CI = –8.35, –5.05) compared to GCSE English (–5.84 GPD, 95% CI = –7.18, –4.50) were very similar in the full model. The greatest attenuation occurs from the addition of weekly cannabis use in the child behaviour model and previous education (Tables S6–S9). After attenuation there is still evidence of a moderate effect of tobacco use on education at age 16.

**Table 2:** The association of GCSE English with adolescent substance use including different imputed covariate models.

*Exposure*		*Univariable*		*Adjusted 1*		*Adjusted 2*		*Adjusted 3*		*Fully Adjusted*	
		*GPD*	*95% CI*	*GPD*	*95% CI*	*GPD*	*95% CI*	*GPD*	*95% CI*	*GPD*	*95% CI*
Cannabis use	Never	–	–	–	–	–	–	–	–	–	–
*n = 4375*	Ever	–2.80	–3.71, –1.90	–2.41	–3.32, –1.50	–2.53	–3.35, –1.70	–2.38	–3.01, –1.76	–0.59	–1.44, 0.28
Cannabis status	None	–	–	–	–	–	–	–	–	–	–
*n = 4375*	Non–weekly	0.41	–1.15, 1.97	0.61	–0.96, 2.17	–0.21	–1.63, 1.21	–1.37	–2.44, –0.29	0.72	–0.45, 1.90
	Weekly	–5.93	–8.34, –3.53	–5.70	–8.10, –3.30	–4.51	–6.68, –2.34	–4.33	–5.95, –2.70	–2.09	–3.79, –0.40
CAST score 4+	No	–	–	–	–	–	–	–	–	–	–
*n = 4087*	Yes	–5.15	–7.69, –2.60	–4.86	–7.40, –2.32	–4.16	–6.47, –1.85	–3.24	–4.99, –1.49	–1.35	–3.15, 0.46
Smoking status	None	–	–	–	–	–	–	–	–	–	–
*n = 3760*	Non–daily	–2.80	–4.06, –1.53	–2.62	–3.88, –1.36	–2.87	–4.02, –1.73	–2.44	–3.30, –1.58	–1.96	–3.01, –0.90
	Daily	–11.90	–13.47, –10.33	–10.79	–12.37, –9.21	–10.04	–11.49, –8.58	–6.49	–7.59, –5.39	–5.84	–7.18, –4.50
Cotinine smoking status	Non–Smoker	–	–	–	–	–	–	–	–	–	–
*n = 2827*	Smoker	–10.72	–12.42, –9.03	–9.77	–11.49, –8.06	–8.42	–9.98, –6.86	–5.15	–6.32, –3.97	–4.37	–5.63, –3.11

Educational outcome here was English General Certificate of State Education (GCSE) results, which were derived from median scores within grade boundaries; four covariate models have been used and each builds on the previous model. These models are grouped into proximal and distal determinants. Adjustment 1 model includes binary maternal substance use behaviours (mother smokes, mother binge drinks and mother uses cannabis). Adjustment 2 model included demographics [socio‐economic status (SES), maternal education and income] and sex. Adjustment 3 model includes the individuals previous education before substance use is more likely [standard deviation (SD) change for Key Stage 2 English and Mathematics]. The fully adjusted model includes child's substance use behaviour measured at the same time as the exposure (child drinking, child weekly cannabis use for the tobacco smoking exposures and child smoking for the cannabis use exposures) and conduct disorder. Covariates were imputed to increase *n*. All the *testparm* results were *P* < 0.001. GPD = grade point difference. CAST = Cannabis Abuse Screening Test; CI = confidence interval.

**Table 3:** The association of GCSE mathematics with adolescent substance use including different imputed covariate models.

*Exposure*		*Univariable*		*Adjusted 1*		*Adjusted 2*		*Adjusted 3*		*Fully Adjusted*	
		*GPD*	*95% CI*	*GPD*	*95% CI*	*GPD*	*95% CI*	*GPD*	*95% CI*	*GPD*	*95% CI*
Cannabis use	Never	–	–	–	–	–	–	–	–	–	–
*n = 4264*	Ever	–5.42	–6.52, –4.32	–4.78	–5.88, –3.68	–4.90	–5.92, –3.88	–4.31	–5.10, –3.53	–2.43	–3.53, –1.34
Cannabis status	None	–	–	–	–	–	–	–	–	–	–
*n = 4264*	Non‐weekly	–3.83	–5.73, –1.93	–3.33	–5.23, –1.43	–4.01	–5.76, –2.25	–4.66	–6.01, –3.30	–2.15	–3.64, –0.65
	Weekly	–6.91	–9.92, –3.89	–6.21	–9.22, –3.19	–6.05	–8.82, –3.27	–6.05	–8.18, –3.92	–3.37	–5.59, –1.15
CAST score 4+	No	–	–	–	–	–	–	–	–	–	–
*n = 3978*	Yes	–8.88	–11.98, –5.78	–8.05	–11.15, –4.95	–7.89	–10.77, –5.01	–6.38	–8.59, –4.16	–3.52	–5.81, –1.23
Smoking status	None	–	–	–	–	–	–	–	–	–	–
*n = 3658*	Non‐daily	–5.55	–7.09, –4.02	–5.28	–6.81, –3.76	–4.62	–6.04, –3.20	–3.61	–4.71, –2.52	–1.78	–3.10, –0.45
	Daily	–16.72	–18.57, –14.86	–15.26	–17.15,–13.38	–13.37	–15.15, –11.59	–9.07	–10.43, –7.71	–6.70	–8.35, –5.05
Cotinine smoking status	Non‐smoker	–	–	–	–	–	–	–	–	–	–
*n = 2747*	Smoker	–15.42	–6.51, –4.32	–13.77	–15.81,–11.72	–11.67	–13.57, –9.77	–7.74	–9.19, –6.29	–6.19	–7.75, –4.62

The educational outcome here was Mathematics General Certificate of State Education (GCSE) results which were derived from median scores within grade boundaries; four covariate models has been used and each builds on the previous model. These models are grouped into proximal and distal determinants. Adjustment 1 model includes binary maternal substance use behaviours (mother smokes, mother binge drinks and mother uses cannabis). Adjustment 2 model included demographics [socio‐economic status (SES) maternal education and income] and sex. Adjustment 3 model includes the individuals previous education before substance use is more likely [standard deviation (SD) change for Key Stage 2 English and Mathematics]. The fully adjusted model includes child's substance use behaviour measured at the same time as the exposure (child drinking, child weekly cannabis use for the tobacco smoking exposures and child smoking for the cannabis use exposures) and conduct disorder. Covariates were imputed to increase *n*. All the *testparm* results were *P* < 0.001. GPD = grade point difference. CAST = Cannabis Abuse Screening Test; CI = confidence interval.

**Table 4:** The association of non‐completion of five or more GCSEs with adolescent substance use including different imputed covariate models.

*Exposure*		*Univariable*		*Adjusted 1*		*Adjusted 2*		*Adjusted 3*		*Fully adjusted*	
		*OR*	*95% CI*	*OR*	*95% CI*	*OR*	*95% CI*	*OR*	*95% CI*	*OR*	*95% CI*
Cannabis use	Ever	–	–	–	–	–	–	–	–	–	–
*n = 4436*	Never	1.76	1.52, 2.05	1.66	1.42, 1.94	1.79	1.51, 2.12	2.13	1.73, 2.61	1.43	1.06, 1.91
Cannabis status	None	–	–	–	–	–	–	–	–	–	–
*n = 4436*	Non‐weekly	1.06	0.80, 1.40	1.01	0.76, 1.34	1.11	0.81, 1.50	1.57	1.08, 2.28	0.92	0.61, 1.38
	Weekly	2.74	1.93, 3.88	2.63	1.84, 3.77	2.57	1.74, 3.80	3.25	2.03, 5.22	1.90	1.16, 3.12
CAST score 4+	No	–	–	–	–	–	–	–	–	–	–
*n = 3561*	Yes	3.01	2.07, 4.37	2.84	1.93, 4.17	2.95	1.93, 4.51	3.40	2.02, 5.72	2.12	1.22, 3.67
Smoking status	None	–	–	–	–	–	–	–	–	–	–
*n = 3816*	Non‐daily	1.43	1.14, 1.80	1.39	1.11, 1.76	1.48	1.15, 1.90	1.58	1.16, 2.15	1.22	0.84, 1.78
	Daily	7.14	5.59, 9.11	6.32	4.92, 8.12	6.96	5.27, 9.20	6.72	4.81, 9.39	4.75	3.11, 7.27
Cotinine smoking status	Non‐Smoker	–	–	–	–	–	–	–	–	–	–
*n = 2870*	Smoker	5.15	3.99, 6.66	4.59	3.53, 5.98	4.36	3.27, 5.80	4.05	2.86, 5.73	3.47	2.36, 5.10

The educational outcome here was the binary non‐completion of 5+ General Certificates of State Education (GCSEs) at grade C or above. Four covariate models have been used and each builds on the previous model. These models are grouped into proximal and distal determinants. Adjustment 1 model includes binary maternal substance use behaviours (mother smokes, mother binge drinks and mother uses cannabis). Adjustment 2 model included demographics [socio‐economic status (SES), maternal education and income] and sex. Adjustment 3 model includes the individuals previous education before substance use is more likely [standard deviation (SD) change for Key Stage 2 English and Mathematics]. The fully adjusted model includes child's substance use behaviour measured at the same time as the exposure (child drinking, child weekly cannabis use for the tobacco smoking exposures and child smoking for the cannabis use exposures) and conduct disorder. Covariates were imputed to increase *n*. All the *testparm* results were *P* < 0.001. CAST = Cannabis Abuse Screening Test; CI = confidence interval; OR= odds ratio.

**Table 5:** The association of school dropout with adolescent substance use including different imputed covariate models.

*Exposure*		*Univariable*		*Adjusted 1*		*Adjusted 2*		*Adjusted 3*		*Fully Adjusted*	
		*OR*	*95% CI*	*OR*	*95% CI*	*OR*	*95% CI*	*OR*	*95% CI*	*OR*	*95% CI*
Cannabis use	Ever	–	–	–	–	–	–	–	–	–	–
*n = 4434*	Never	2.79	1.70, 4.57	2.48	1.49, 4.12	2.55	1.53, 4.24	2.47	1.45, 4.19	2.75	1.18, 6.39
Cannabis status	None	–	–	–	–	–	–	–	–	–	–
*n = 4434*	Non‐weekly	1.98	0.89, 4.42	1.80	0.80, 4.06	1.94	0.86, 4.41	2.48	1.05, 5.84	1.96	0.74, 5.21
	Weekly	6.83	3.37, 13.85	6.24	3.02, 12.92	5.94	2.83, 12.45	5.23	2.33, 11.72	4.44	1.79, 11.00
CAST score 4+	No	–	–	–	–	–	–	–	–	–	–
*n = 4137*	Yes	8.15	4.00, 16.60	7.19	3.43, 15.08	7.23	3.39, 15.42	5.41	2.40, 12.20	4.40	1.70, 11.38
Smoking status	None	–	–	–	–	–	–	–	–	–	–
*n = 3814*	Non‐daily	0.90	0.31, 2.58	0.86	0.30, 2.48	0.86	0.30, 2.50	0.86	0.29, 2.55	0.73	0.20, 2.66
	Daily	7.55	4.23, 13.47	6.48	3.49, 12.04	6.17	3.29, 11.56	4.78	2.45, 9.35	2.23	1.11, 9.39
Cotinine smoking status	Non‐Smoker	–	–	–	–	–	–	–	–	–	–
*n = 2869*	Smoker	5.44	2.85, 10.36	5.03	2.56, 9.88	4.48	2.26, 8.90	3.27	1.59, 6.70	2.54	1.12, 5.74

The educational outcome here was school dropout, which was derived from not gaining any General Certificate of State Education (GCSE) grades or receiving only U grades (graded as unclassified); four covariate models were used and each builds on the previous model. These models are grouped into proximal and distal determinants. Adjustment 1 model includes binary maternal substance use behaviours (mother smokes, mother binge drinks and mother uses cannabis). Adjustment 2 model included demographics [socio‐economic status (SES), maternal education and income] and sex. Adjustment 3 model includes the individuals previous education before substance use is more likely [standard deviation (SD) change for Key Stage 2 English and Mathematics]. The fully adjusted model includes child's substance use behaviour measured at the same time as the exposure (child drinking, child weekly cannabis use for the tobacco smoking exposures and child smoking for the cannabis use exposures) and conduct disorder. Covariates were imputed to increase *n*. All the *testparm* results were *P* < 0.001. CAST = Cannabis Abuse Screening Test; CI = confidence interval; OR= odds ratio.

### Influence of imputation

The results presented are based on the imputed data set. We have compared the imputed results with the complete case for the self‐report substance use and for the biomarker, cotinine, in order to determine the consistency of the imputed results; there were no major differences (Tables S3–S4, see Appendix 2).

## Discussion

### Main findings and implications

Cannabis use by young people by age 15 was associated consistently with poorer performance across a range of objective indicators of subsequent educational attainment. This association was attenuated, but remained apparent following adjustment for a wide range of possible confounding factors. Further adjustment for educational attainment prior to cannabis use led to further attenuation. In general, higher cannabis use was associated with lower attainment. Using cannabis was associated with a GCSE score reduction of approximately 5%, which is half a grade. The association was similar in girls compared to boys. In some instances the effects of tobacco seemed stronger and more substantial. Higher tobacco use was generally associated with poorer outcomes, and effects in girls were similar to those in boys. The effects of biologically verified tobacco use were very similar to those of self‐reported tobacco use.

Our results are broadly consistent with other evidence suggesting the adverse effect of cannabis use on subsequent educational performance 2, 19, 20, 21, 22, 23. Other studies in general have not considered both cannabis and tobacco use by young people and subsequent educational outcomes in the same cohort; rather, they have reported the effects of cannabis use adjusted for tobacco use. A small number of previous studies have considered the effects of tobacco use on educational attainment, and have reported similar patterns of association to those that we observed 24, 25, 26, 27. The associations for cannabis were found to be non‐specific; due to tobacco use by age 15 showing very similar patterns of association with the same educational outcomes.

Associations of biologically verified tobacco use were similar to those of the nearest equivalent self‐reported exposure (daily smoking), suggesting that reporting bias had not substantially influenced the latter. Few studies have investigated the specific effect of school‐based outcome data with substance use, rather than self‐reported education variables 19, 20, 28. The heterogeneity between current studies’ measures of education and of substance use allows for little statistical comparison between studies.

Previous studies have not attempted to adjust for as comprehensive a range of confounding factors as we included in our multivariable analyses 1, 19, 28. Adjustment for these factors considerably attenuated our estimates of effects of cannabis use. Specific adjustment of results from the covariates within each model was also investigated (Tables S6–S9). Adjustment for each covariate individually attenuated estimates to a similar extent. The highest attenuation occurred within the ‘prior educational attainment’ model and the ‘child behaviour’ model. The size of the reduction of the association is similar, with a greater than twofold reduction, which in our analyses still provides evidence of a moderate effect of daily tobacco/weekly cannabis use on educational performance. In general, following such adjustment, the association of tobacco use on educational attainment appeared stronger and of greater magnitude than those of cannabis use; the exception to this pattern was in relation to the association on ’school‐dropout’, wherein the fully adjusted analyses association of cannabis use appeared stronger and of greater magnitude than those of tobacco use.

In the ’child behaviour’ model, the largest attenuation occurs from the co‐administration of cannabis or tobacco on tobacco or cannabis use, respectively. Demographics appear to attenuate the relationship between substance use exposure and educational attainment in a similar pattern throughout. Consideration of month of birth and maternal education also led to attenuation of the estimates. We observed an association of sex only on frequent cannabis and tobacco use, with the odds of frequently using cannabis being higher for males and the odds of frequently using tobacco being higher for females. There is also an association of sex on education variables, with males having a reduced GCSE English GPD, having higher odds of achieving five or more GCSEs but also having higher odds of being a school dropout.

We have attempted to control for reverse causation by including a measure of educational attainment prior to the initiation of cannabis use in our model. We observed an association between previous education and substance use when considered separately (Table S1). This adjustment led to attenuation of the effects of cannabis use suggesting that reverse causation, i.e. prior educational difficulties predisposing to cannabis use, was an issue in our study population. This could be attributed to a third factor, such as clustered behaviours within peer groups.

### Strengths and limitations

The strengths of the present study include its general population basis and prospective design and also the availability of extensive prospective measures of relevant covariates, the availability of a biologically verified measure of tobacco use and the availability of objective measures of key dimensions of educational attainment obtained through record linkage. This linkage also allowed us to consider the effects of cannabis use on specific aspects of educational attainment, such as performance in different subjects, in a way that has not been possible in previous studies 28.

The study also has limitations. First, the ALSPAC cohort is subject to loss to follow‐up at each stage. Male cohort members and those from lower socio‐economic status (SES) groups are also less likely to attend assessment interviews. Therefore, not attending the clinic is the largest contributor to missing data. To an extent, we were able to mitigate this problem and any bias that may have resulted from it through multiple imputations; however, the validity of multiple imputations is based on assumptions that are usually impossible to verify 29. There may be greater misclassification of cannabis than tobacco. This is because the sensitivity of questions about substance use and other behaviours means that the participant may decide to withhold certain information by not answering the question or not answering it honestly, for fear that it would be passed on to parents or teachers, thereby causing bias. This would therefore underestimate the number of substance users or misclassify users as non‐users, in turn causing response bias to the results. The cotinine validates the self‐report of tobacco, but there is no equivalent valid biological measure for cannabis. We were unable to measure the effect of peer groups in this study, as delinquent peer groups may have an effect both of substance use and on educational attainment; therefore, there may be unmeasured confounding in our study. Finally, an immunoassay of cotinine has been shown to not be as precise as the gas chromatography–mass spectrometric (GC‐MS) quantitative method for cotinine extraction, possibly creating bias in the measurements 30.

### Conclusions and policy implications

Given these patterns of association and attenuation, alongside the non‐specific nature of the association, our evidence suggests that, rather than being causal, the consistent association between cannabis use by young people and their subsequent poorer educational outcomes is likely to arise through a combination of confounding factors. These factors are related to both the tendency to use psychoactive substances and to perform less well in educational assessments. Alongside this, reverse causation is related to the fact that children who are less successful educationally have a heightened risk of substance use, which could be for several reasons. It is not possible to discount a causal basis for our findings completely, as both cannabis and tobacco use may influence subsequent educational attainment causally through independent mechanisms. Moreover, the question may not have important implications for policy. There are good reasons to prevent both cannabis and tobacco use by young people related to the effects of smoking on cardiorespiratory health and because the former, as it is illegal, exposes young people to risk of criminalization. Our findings, however, which suggest that prevention of cannabis use may improve educational outcomes in young people, particularly the socially disadvantaged, is probably unrealistic. It therefore follows that other interventions are likely to be needed to achieve this important policy objective.
